# Protective Effects of *Cyperus Rotundus* Extract on Amyloid β-Peptide (1-40)-Induced Memory Impairment in Male Rats: A Behavioral Study

**DOI:** 10.18869/nirp.bcn.8.3.249

**Published:** 2017

**Authors:** Mehdi Mehdizadeh, Fataneh Hashem Dabaghian, Asie Shojaee, Nima Molavi, Zahra Taslimi, Ronak Shabani, Sara Soleimani Asl

**Affiliations:** 1. Research Institute for Islamic and Complementary Medicine, Iran University of Medical Sciences, Tehran, Iran.; 2. Department of Medicine, School of Medicine, Iran University of Medical Sciences, Tehran, Iran.; 3. Neurophysiology Research Center, Hamadan University of Medical Sciences, Hamedan, Iran.; 4. Department of Anatomy, School of Medicine, Iran University of Medical Sciences, Tehran, Iran.; 5. Endometrium and Endometriosis Research Center, Hamedan University of Medical Sciences, Hamedan, Iran.

**Keywords:** Alzheimer disease, Amyloid β-peptide, Cyperus rotundus, Spatial memory

## Abstract

**Introduction:**

The Alzheimer Disease (AD) is the most common form of dementia that leads to memory impairment. As the oxidative stress plays an important role in AD pathogenesis, the current study aimed at examining the protective effects of *Cyperus Rotundus* as an antioxidant on amyloid β (Aβ)-induced memory impairment.

**Methods:**

Twenty-eight Wistar male rats received intrahippocampal (IHP) injection of the Aβ (1-40) and *C. rotundus* (400 mg/kg, intraperitoneally). Spatial memory was assessed by the Morris water-maze (MWM) task.

**Results:**

In the MWM, Aβ (1-40) significantly increased escape latency and traveled distance (P<0.001). The administration of *C. rotundus* attenuated the Aβ-induced memory impairment in the MWM task.

**Conclusion:**

The current study findings showed that *C. Rotundus* could improve the learning impairment, following the Aβ treatment, and it may lead to an improvement of AD-induced cognitive dysfunction.

## 1. Introduction

The Alzheimer Disease (AD) is a progressive and irreversible loss of neurons commonly characterized by a gradual decline of memory, language, and cognitive ability (Kumar, Shankar, Reddy, Kumar, & Sumalatha, 2014). Accumulation of Amyloid β (Aβ) in the brain is considered as 1 of the major contributing factors to the development of AD (Citron et al., 1997). Nabeshima and Nitta reported that intraventricular administration of Aβ in the rats resulted in learning and memory impairment accompanied by a decrease in choline acetyltransferase activity, suggesting that accumulation of Aβ is related to cognitive deficiency in AD (Nabeshima & Nitta, 1994). Administration of Aβ leads to changes in Long-Term Potentiation (LTP) in the hippocampus and, consequently, impairs cognition and memory in rodents (Shankar et al., 2008). Stress oxidative, protein oxidation, Reactive Oxygen Species (ROS) formation, and subsequent neuronal death are reported following Aβ deposition generated by an imbalance between ROS and internal antioxidants. Evidence show that appropriate nourishing with external antioxidants could improve brain damage and cognitive function (Khodamoradi, Komaki, Salehi, Shahidi, & Sarihi, 2015; Nezhadi et al., 2011).

Furthermore, the external antioxidants can prevent the detrimental consequences of Aβ and are considered as a promoting approach to neuroprotection in the AD brain (Ghahremanitamadon et al., 2014; Zargooshnia et al., 2014). *Cyperus rotundus*, as a species of sedge and traditional medicine are widely used worldwide to treat stomach ailments and wounds (Puratchikody, Devi, & Nagalakshmi, 2006; Uddin, Mondal, Shilpi, & Rahman, 2006).

It is shown that the major chemical components of *C. rotundus* are essential oils, flavonoids, terpenoids, mono and sesquiterpenes (Singh, Kulshrestha, Gupta, & Bhargava, 1970; Sonwa & Knig, 2001). Also, it is shown that hydroalcoholic extract of *C. rotundus* exhibited powerful free radical scavenging, especially against *1,1-diphenyl-2-picryl-hydrazyl (DPPH)* and superoxide anions, as well as a moderate effect on nitric oxide (Yazdanparast & Ardestani, 2007). This extract showed an inhibitory effect against lipid peroxidation, protein oxidation, and glycoxidation (Bahramikia, Ardestani, & Yazdanparast, 2009; Kilani et al., 2008). It appears that *C. rotundus* contains potent components such as flavonoids that may be potentially useful to modulate the immune cell functions, provoking analgesics, and defending against inflammation and stress oxidative (Dhillon, Singh, Kundra, & Basra, 1993). Malekian and Ghannadi showed that administration of *C. rotundus* improved the scopolamine-induced learning and memory deficit in mice (Malekian, Rabbani, & Ghannadi, 2012). Due to the common usage of traditional medicine and natural antioxidants, the current study aims at investigating the ameliorating effects of *C. rotundus* extracts on Aβ (1-40)-induced amnesia.

## 2. Methods

### 2.1. Materials

The Aβ (1–40) was purchased from sigma-Aldrich company (St. Louis, MO, USA). Aβ 1-40 was solubilized in sterile water at 1 μg/μL concentration and stored at −20°C until use.

### 2.2. Animals

Adult male Wistar rats (Pasteur Institute, Tehran, Iran), weighed 250 to 300 g were used in the study. The rats were accommodated the animal house in a 12:12 hours light/dark cycle (light on, 7:00 AM; light off, 7:00 PM) with free access to food and water. A week before the experimental procedure, the animals were habituated to their new environment. The guidelines of the National Institute of Health Guide for Care and Use of Laboratory Animals were performed for all experiments, and approved by the Veterinary Ethics Committee of the Iran University of Medical Sciences, Tehran, Iran.

The animals were randomly classified into the following groups (n=7 each group): The control group which was left undisrupted; and The sham-operated group. The Aβ model group received single bilateral intrahippocampal (IHP) injections of 6 μg Aβ 1-40 (Zargooshnia et al., 2014). The *Cyperus rotundus*-treated group received intraperitoneal injection of *C. rotundus* extract (400 mg/kg) following IHP injection of Aβ 1-40 for 14 days (Hsieh, Peng, Wu, & Wang, 2000).

### 2.3. Preparation of *C. rotundus* extract

In June 2014, the dried *C. rotundus*, with herbarium code TARI 12569, was collected from Iranian Institute of Medicinal Plant Field and grounded into coarse powder by electrically driven device and was soaked into aqueous ethanol (80%) for 1 week. It was passed through a Whatman filter paper (a cellulose filter to specify and recognize the materials in the qualitative analytical techniques) and vaporized by a rotary evaporator under the reduced pressure at a maximum of 40°C.The extract was completely dissolved in distilled water and kept at 4°C (Malekianet et al., 2012).

### 2.4. Stereotaxic surgery

**Figure 1.**
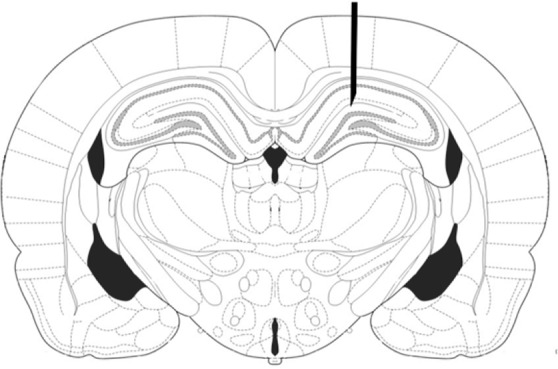
Schematic photograph representing the microinjection site of Amyloid β into the hippocampus (black arrow).

The previously established method of stereotaxic surgery was used (Zargooshnia et al., 2014) In brief, rats were anesthetized by xylazine (10 mg/kg) and ketamine (100 mg/kg), and placed into a stereotaxic device (Stoelting, USA). After retraction of scalp, the area surrounding bregma was cleaned and dried. Relative to the bregma and with the stereotaxic arm at 0°, the coordinates for the dentate gyrus were anterior-posterior (AP): 3.6 mm from bregma; Mediolateral (ML): +2.3 mm from mid-line; Dorsoventral (DV): 3 mm from skull surface (Figure 1). Aβ solution (6 μL) was bilaterally injected into the region using a 10 μL microsyringe (Hamilton-Reno, NV, USA). Sham operated rats received vehicle solution.

### 2.5. Assessment of spatial memory

Morris Water-Maze (MWM) is a behavioral task to assess hippocampal-related learning, including acquisition and retention of spatial memory and plays an important role in the validation of rodent models for neurocognitive disorders such as the Alzheimer disease (D’Hooge & De Deyn, 2001; Morris, Garrud, Rawlins, & O’Keefe, 1982). Therefore, the protocol used in the current investigation was derived from the previously performed study Soleimani Asl et al. (2013). In brief, a black circular pool filled with water (22±1°C) with an invisible plexiglass platform (located 1 cm below the water) was used. Some constant visual cues such as table, library, and computer were located around the MWM room.

The North, East, South, and West locations were selected to start the training trials. The animals were trained for 3 consecutive days at the same time (10:00 to 12:00 AM). Training included 2 blocks with 4 trials. There was a 5-minute rest between consecutive blocks. During trials, from each of the starting positions, the animals swam up the located hidden platform. The animals were allowed to spend 30 seconds on the platform between the 2 trials. There was a video camera above the pool that recorded escape latency (the time taken to reach the hidden platform) and traveled distance (the length of the swim path). On day 4, a probe trial was performed in which the platform was removed from the pool and each rat was allowed to swim for 60 seconds and percentage of entrance into the target quadrant was recorded.

### 2.6. Statistical analysis

Data were expressed as mean±standard error of the mean (SEM) and processed by commercially available software SPSS version 16. Results were analyzed using repeated measure and 2 way analysis of variance (treatment and training days as the factors). The Turkey multiple comparison test was used to analyze the significance of the differences between the groups, when appropriate. P<0.05 was considered statistically significant.

## 3. Results

**Figure 2.**
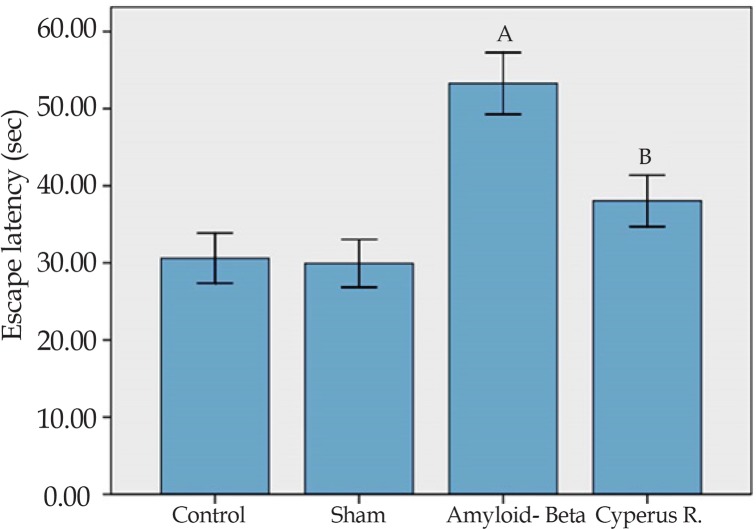
The mean of latencies to find hidden platform in the MWM. Each block represents the average latency of three consecutive trial days. Data present as mean±S.E.M. (a: P<0.001 vs. control and sham groups; b: P<0.05 vs. Aβ-treated group).

Effects of *Cyperus rotundus* on the Aβ (1-40)-induced increase in escape latency in Morris water-maze: A 2-way analysis of variance of escape latency revealed significant effects of treatment [F(3, 4233)=10.63, P<0.001]. In addition, there was no significant interaction between treatment and training days. As shown in Figure 2, escape latency (the time to find the hidden platform) was less in the control group than the other groups. More time to find the hidden platform indicates more intense spatial memory impairment. A post hoc analysis of the 3 training days showed a significant difference between the control and sham-operated groups, and the rats that received Aβ (P<0.001). According to the current study results, the administration of *C. rotundus* caused significant reduction in escape latency compared with the Aβ-treated group (P<0.05).

**Figure 3.**
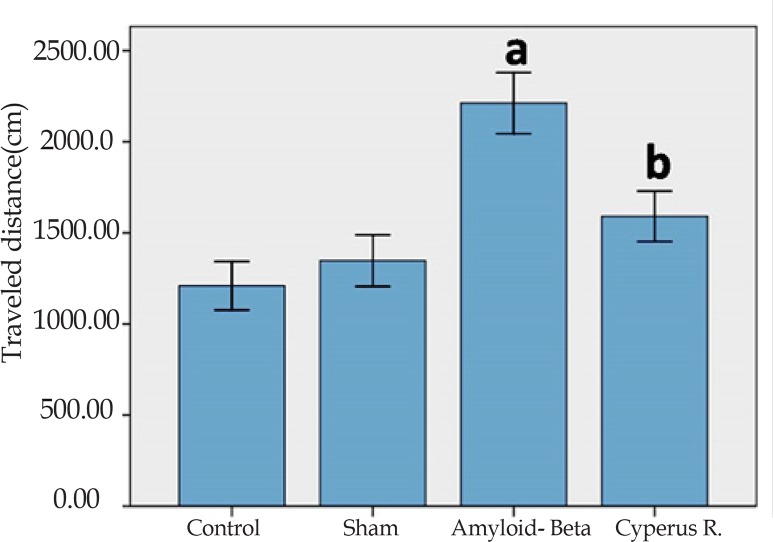
The mean of traveled distance in the MWM. Each block represents the average of traveled distance of four consecutive trial days. Data present as mean±S.E.M. (a: P<0.001 vs. control and sham groups; b: P<0.05 vs. Aβ-treated group).

Effects of *Cyperus rotundus* on the Aβ (1-40)-induced increase in traveled distance in Morris water-maze: In accordance with the latency data, treatment had significant effect [F(3, 7080)=9.83, P<0.001]. There was no significant effect in both training days, and also no significant difference was observed in the interaction between training days and treatment. A significant difference in traveled distance was observed between Aβ-treated rats with the control and sham-operated groups (P<0.001) (Figure 3). Aβ-treated rats that received *C. rotundus* extract for 7 days showed less traveled distance, in comparison with the Aβ-treated group (P<0.05).

**Figure 4.**
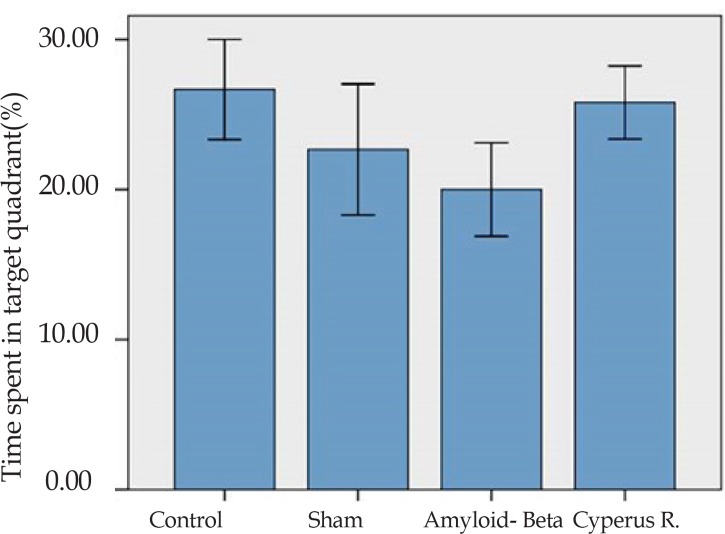
The mean of the percent of time spent target quarter in the probe trial in the MWM. Data present as mean±S.E.M.

Effects of *Cyperus rotundus* on the Aβ (1-40)-induced reduction in time spent in target quadrant in Morris water-maze: As shown in Figure 4, a 1-way analysis of variance (ANOVA) of time spent percent in the target quadrant showed that the control group spent more time in target quadrant (26.66±3.33) than the sham-operated (22.66±4.37), Aβ-treated (20±3.1) and *C. rotundus* (25.8±2.43).

## 4. Discussion

The current study aimed at evaluating the protective effects of *C. rotundus* extract on memory impairment following intrahippocampal injection of Aβ (1-40). The major findings of the current study were: (a) Intrahippocampal injection of Aβ (1-40) resulted in learning impairment; (b) Treatment with *C. rotundus* extract was protective against Aβ-induced memory impairment.

Previously published experimental studies reported that the infusion of Aβ(1–40) into the brain impaired one-trial/day reward learning (Malin et al., 2001), and memory in radial-arm maze task confirmed the current study results (Malin et al., 2001; Tanaka et al., 1998). As memory impairment and degeneration of cholinergic neurons can be observed following the deposition of β-amyloid protein in the brain; it seems that the β-amyloid-treated rats could be used as the animal model for the Alzheimer disease (Nitta, Itoh, Hasegawa, & Nabeshima, 1994).

It seems that due to the low antioxidant and cell membrane lipid, the brain is sensitive to oxidative stress (Butterfield & Lauderback, 2002). Therefore, employment of external antioxidants, such as various spices and herbs, can be 1 of the popular remedial strategies to treat neurological diseases, and recovery of brain damage and cognitive deficiency (Azad, Rasoolijazi, Joghataie, & Soleimani, 2011; Ghahremanitamadon et al., 2014).

*Cyperus rotundus* is a traditional medicinal plant used against stomach disorders and inflammatory bowel diseases (Jagtap, Shirke, & Phadke, 2004; Uddin et al. 2006).The current study demonstrated that administration of *C. rotundus* extract could improve Aβ-induced memory impairment. The protective effect of *C. rotundus* extract on memory can be related to its function of scavenging free radicals (Yazdanparast & Ardestani, 2007).

Ardestani et al., showed the preventing effects of *C. rotundus* on oxidative protein damages by decreasing oxidative stress (Ardestani & Yazdanparast, 2007). Anti-oxidant activity and metal chelating properties of *C. rotundus* are shown by Fluorescence Recovery After Photobleaching (FRAP) and Trolox Equivalent Antioxidant Capacity (TEAC) assays (Ardestani & Yazdanparast, 2007). This herbal extract with anti-inflammatory properties is safe in animals (Dang, Parekar, Kamat, Scindia, & Rege, 2010; Seo et al., 2001).

It seems that the antioxidant properties of *C. rotundus* are due to its phenolic content (Ardestani & Yazdanparast, 2007), which should be studied further. In conclusion, intrahippocampal injection of Aβ induced significant learning deficits in Morris water-maze tasks, and *C. rotundus* treatment improved Aβ-induced deficiencies. Therefore, it is likely that *C. rotundus* may be useful to treat patients with impaired memory function.
